# 3D printing technology and internet of things prototyping in family practice: building pulse oximeters during COVID-19 pandemic

**DOI:** 10.1186/s41205-020-00086-1

**Published:** 2020-11-02

**Authors:** Matteo Capobussi, Lorenzo Moja

**Affiliations:** 1 Department of Biomedical Sciences for Health, University of Milan, Milan, Italy; 2 Studio Medico Associato, Viale Rimembranze 5, 20844 Triuggio, MB Italy

**Keywords:** Covid19_1_, Pulse Oximeter_2_, Internet of Things_3_, Maker Culture_4_, Medically underserved Area_5_

## Abstract

Family doctors can have an active role in identifying significant population needs and solutions. During the COVID-19 epidemic, patient home monitoring with pulse oximetry has been a key aspect of care of patients. However, pandemics bring shortage of medical equipment such as pulse oximeters. Through the local maker community, in a matter of days four “smart” pulse oximeters were built. Following Internet of Things principles, the prototypes were programmed to transmit real-time data through Wi-Fi directly to the doctors. Each pulse oximeter served a family doctor during the pandemic. In this article we describe the process that led to the production of the technology and provide detailed instructions, which have also been shared in maker-oriented websites. Dissemination can potentially lead to additional small-scale productions, limiting future shortages.

**Supplementary Information:**

The online version contains supplementary material available at 10.1186/s41205-020-00086-1.

## Introduction

Family Medicine aims to deliver new medical discoveries associated with important benefits to the general population, closing the gap between research and standard practice. Most biomedical research transferred at community level encompassed medicines and assistive technologies, with family medicine being the last receiver of innovation [1]. However new frontiers are opening up. The Internet of Things (IoT) is a system of smart devices connected to the Internet and communicating over a network [2]. In this context family medicine per se can become the starting point of innovative approaches.

The maker culture is a movement identifying people devoted to the creation of new devices and tinkering with existing ones, usually involving electronics and software programming [3]. Makers often aggregate in so called fabrication laboratories (often called fab labs) where experts in multiple technologies try to leverage their knowledge and skills [4]. Mastering technologies such as 3D printing [5, 6], micro-soldering, electronic circuit design and production, and software programming is a key feature of this new generation of workers. Therefore, labs are spaces for innovation equipped with 3D printers, laser cutters, chemical reagents, and other machines with the capability of starting small-scale production of smart devices. The overall potential of these fab labs in family medicine practice is unknown, but their role in the COVID-19 pandemic has been substantial [7]. The purpose of this project is to describe our experience in the assembly and implementation of an open source pulse oximeter as a stop-gap medical device during a peak phase of the COVID-19 pandemic in Milan, an Italian city in one of the hardest-hit geographical area by the pandemic.

## Methods

### The problem

The importance of pulse oximetry has dramatically risen during COVID-19 epidemic. Desaturation is often the only parameter signaling the initial stages of the COVID-19 interstitial pneumonia; it is an essential sign that a patient is clinically deteriorating. Pulse oximetry is essential to monitor patients and serves as a quantitative metric for patients who need hospitalization [8]. It is also non-invasive and easy to use. Home care benefits from telemonitoring with pulse oximeters, especially in times when a pandemic from a deadly and highly contagious virus suggests avoiding unnecessary home visits [9].

During the first weeks of pandemic, Italian general practitioners faced an immediate shortage of pulse oximeters. Medical equipment vendors as well as pharmacies quickly ran out of all models. Even online shops estimated delivery times greater than 1 month. Most attempts to procure these devices failed, leaving patients and family doctors in need of home monitoring without them.

### The assembly of an open source pulse oximeter

**Fig. 1:**
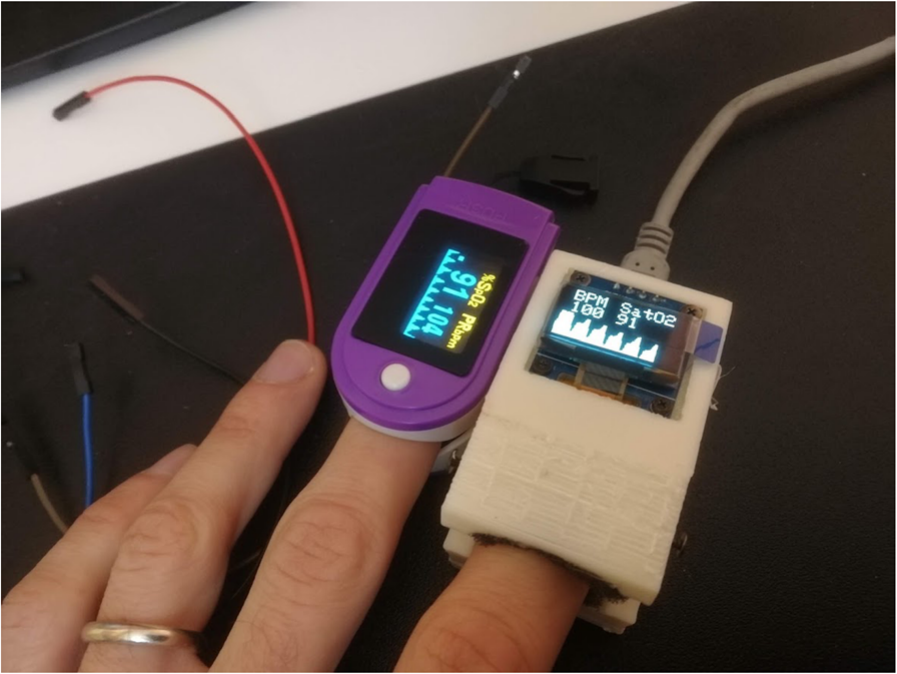
Comparison between a home-made pulse oximeter against a certified one

Using our experience in digital modelling and fabrication, we researched available literature on the topic, ranging from academic studies on the algebraic calculations needed to measure saturation, to do-it-yourself websites reporting instructions for assembling electronic components [10, 11]. Free literature contained detailed background information to support the development of each piece of technology. Using an Arduino Nano, later upgraded to an ESP32 prototyping board for the need of improved calculation capabilities, we assembled the electronics, which included sensor MAX30102 from Maxim Integrated and Adafruit SSD1306 OLED graphic display. All the circuits were then powered by a single 5 V power bank. We were able to design a working 3D printed shell in a matter of hours, using basic shapes available in the Tinkercad free online software tool from Autodesk, Inc., San Rafael, California, USA. In our scenario, timing was essential. 3D printing technology was vital to quickly provide a fitting shell for the electronics, insulating it from external light and thus ensuring the quality of the readings. Having an easy way to adapt spring tension was also important for obtaining reliable measurements. The smallest blood vessels are prone to closure by excessive pressure, while an insufficient compression could facilitate motion artifacts as well as light infiltrations. Our design included insertion points for multiple types of springs, ranging from extension coils to ball pen springs, in order to facilitate replication of our design by other Makers and fab labs. Open source libraries were implemented to add Wi-Fi capability. Specifically, we adapted Hieromon’s Autoconnect library [12]. In fact, we anticipated that it was in the best interest of the patient to have a device automatically transferring information on saturation by email to the family doctor, in addition to showing the same information on a small screen embedded in the device. We also anticipated the importance of an adequate calibration. With the help of the fab lab network, a community of people with strong interest in sharing and collaborating on useful contributions to advancement of society, we developed a software for a streamlined oxygen calibration procedure, using a certified commercial pulse oximeter available as the reference standard [13]. Although the embedded Maxim sensor is certified for a tolerance of +/− 2%, calibrating it using as reference a fingertip commercial pulse oximeter, which also has a +/− 2% error, could theoretically cumulate it to 4%. However, observed data originated by our pulse oximeters were consistent with certified oximeters. Initial quality testing included 8 volunteers that showed a difference of 1% or less between our stop-gap produce and the commercial ones. Furthermore, to increase our confidence on device reliability, before delivering the uncertified pulse oximeter to the patients, we re-evaluated each pulse oximeter against a certified oximeter on that patient (Fig. 1). Other local fab labs, a 3D printing industry and individual makers provided feedback for improving the device and made some corrections to our original software.

## Results

**Fig. 2:**
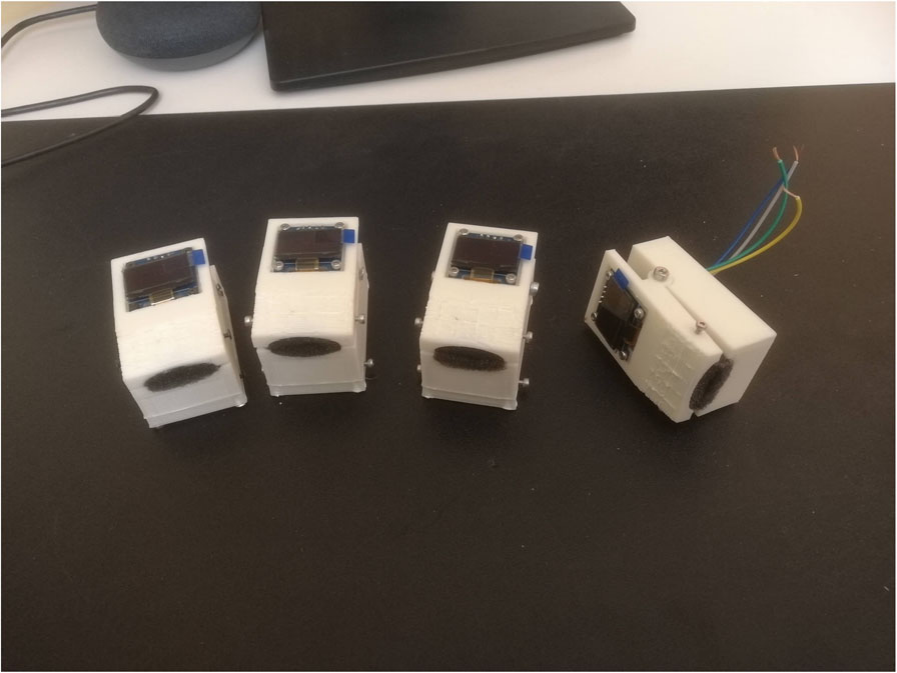
The four pulse oximeters at final stages of production

The entire time to finalize an emergency pulse oximeter was 10 days. Given the novelty of this medical application for medical 3D printing [14], the production capacity, limited resources available and piloting-nature of the solution, the project included after successful creation of 4 pulse oximeters (Fig. 2), one for each general practitioner working in our practice. Doctors agreed that the observed cumulated tolerance of +/− 3% was acceptable given the emergency situation and with respect to the use of the device, which aimed to detect a severe deterioration of respiratory function. They decided subsequently to determine the gravity of patient’s conditions by 2 cutoffs: “normal” above 94%, “intermediate” above 90%, and “severe” defined as 89% and lower. Each family doctor then used the device for the remaining time of the pandemic accordingly to his or her preferences and needs, selecting patients eligible to use the device and benefitting most. Patients were informed of the unavailability of certified devices, and characteristics and limitations of the emergency device, including lack of certification, were carefully illustrated.

After assemblage, in the first 2 weeks, the emergency pulse oximeters assisted six patients. Users were instructed to measure saturation two times a day for 1 weeks. Collected data was automatically sent by email in real-time to the doctors, who called the patients when advice was needed. In the following 4 weeks, 16 more patients were monitored using this device, until “certified” pulse oximeters became available again for purchase. We acknowledge that this small number pales in comparison to the number of patients at home in need of telemonitoring.

We have published free open source detailed building instructions for the making community and those interested, as well as. STL files for 3D printing the shell [13]. Building instruction are also provided in an Additional file 1 (in English) to this article. We disseminated information on the possibility of recurring to this emergency solution until mass production was able again to provide certified pulse oximeters. Despite we were also asked for collaboration and advice to scale up the production, the potential and feasibility of this emergency local solution might be overlooked. Across Italy, many fab labs, enterprises, and charitable organizations reached out to provide support for this initiative for the duration of the crisis.

## Discussion

Pandemics involve the whole world population, bringing shortage of key assets such as medicines or medical equipment. It is of crucial importance that the most up to date medical supporting life technologies are available to everyone who needs them. However, industrial production may need time to adjust to rapidly increasing demand. The world-wide-web has made available scientific knowledge at population level. This may provide an answer to large scale requests. General Practitioners have the capability of swift detection of the population medical needs and can oversee and coordinate the efforts of the local communities to address those needs. However, in order to rapidly finalize open source projects and scale up production of supporting life technologies, as well as deploying devices in different parts of the world, more expertise is needed than that usually available in a fab lab.

Adding a Wi-Fi transmission was a key step in reducing physical contact with an infected patient. This feature is not usually available in commercial pulse oximeters. Following current protocols, for each COVID-19 patient, a nurse with protective gear should daily reach the patient at home in order to measure vital parameters. This could be avoided by developing smart Internet of things medical devices, saving time and reducing exposition to the infection. Privacy issues discouraged us from using web-based data recording and elaboration, therefore email was chosen as the most accessible communication channel.

We acknowledge several limitations. While we attempted to control for variations between patients for example skin tone, differences in blood vessels or finger thickness, these variations may have impacted out tolerance data. Inherent in our project was the fact that our products were not medical devices [15] and were not subject to the same quality and safety standards typically applied [16] when 3D printing is used in the medical sector [17]. As an example, a device either registered or cleared by a regulatory body would have to be waterproof. The temporal need for this testing was openly acknowledged; it simply was not possible with the life and death of patients in the balance of workload among the clinician-investigators. Little time could be afforded to repeated studies of diagnostic accuracy. As a best stop-gap approach, we used a certified sensor for validation of oxygen blood levels until we gained clinical confidence in the device. Against this unique emergency scenario, we favored a precautionary approach, measuring our devices against a certified device at individual level, and limiting the local production of pulse oximeters to few units.

## Conclusion

In times of emergency the ability of prototype development technology to adapt to new needs can be the key for addressing emerging challenges in reasonable times. Solutions can be provided by the industry if they are available. However new technologies can possibly support more delocalized answers. Health professionals, such as family doctors, can have an active role in identifying key needs and developing these potential solutions, which include ex novo fabrication. Advances in medical knowledge and modern technology are the key differences with middle-age epidemics, and we should take advantage of them. The role of local fabrication of smart devices in the context of an emergency should be further studied and supported by dedicated research resources.

## Supplementary Information

**Additional file 1.**

## Data Availability

All data, building instructions, and software, for this study are open source and can be downloaded for free from https://medicitriuggio.altervista.org/come-costruire-in-casa-un-saturimetro/
