# Supplementary material for: 3D printing technology and internet of things prototyping in family practice: building pulse oximeters during COVID-19 pandemic
Source: 3D Print Med. 2020 Nov 2;6:32. doi: 10.1186/s41205-020-00086-1 (PMC7605335; doi:10.1186/s41205-020-00086-1)
Supplement: Supplementary file 1 — Additional file 1. [file 41205_2020_86_MOESM1_ESM.docx]

**Additional file 1 - Pulse oximeter building instructions**

The Arduino sketch, the .stl files required for 3D printing and all the other needed files can be downloaded from here: <https://drive.google.com/open?id=1zvxwnRE-UM87otZBnbQbLkbCB3mnYoJY>

1. List of materials:

• ESP32 card: <https://www.amazon.it/gp/product/B074RGW2VQ/>

• Maxim Max30102 sensor: <https://www.amazon.it/gp/product/B07YXF3L77/>. This sensor requires soldering of both the PINs and a tiny contact on the lower surface. If you are not familiar with micro-welding, we suggest to buy one already soldered

• 0.96 inch I2C LCD display: <https://www.amazon.it/gp/product/B076PDVFQD/>

• 6 M3 15 mm screws

• 2 M3 self-locking nuts

• A spring from a pen

• 12 M2 5 mm screws (optional)

• Rubber band (optional)

• Extension spring (6.25 x 22 mm)

• Dark colored foam, 5 mm thickness

• microUSB cable

• Power Bank (optional)

1. Assembling the pulse oximeter

3D print the external shell parts. When possible, use dark colors to reduce sensor interference from external light. Any plastic (PLA or ABS) can be used, since mechanical stress is not an issue in this scenario. Print the 3 files, orienting them appropriately, remove the support structures at the end of printing. We recommend to set 200 °C for the extruder and 60 °C for the printing surface in PLA prints; 240 °C for the extruder and 100 °C for the surface when using ABS.

Insert the ESP card in the bottom piece with the PINs facing up. Insert the LCD screen in its housing on the top piece. Use 4 M2 screws to secure it. Alternatively, apply some glue, for example with a hot glue gun. The area where the PINs reach through the plastic is the most subject to mechanical stress, so we advise to reinforce it with glue.

Insert the sensor in the housing in the intermediate piece, with the PINs facing inside. Secure it with screws or glue.

In order to shield the sensor from external light, the finger clamp of the oximeter has to be internally covered with foam rubber. Cut two rectangles of foam rubber, then cut an opening for the sensor’s LEDs. Glue the edges of the foam to the printed parts.

Use two M3 screws to join the back of the middle and top pieces. The screws will also hold in place two ballpen springs. For the oximeter to work, it is important to reach a light and constant pressure on the finger. One way to achieve this is to cut a few coils from a ballpoint pen spring, then unwind the two ends for half a centimeter. Insert the extremities in the two small holes on the back of the top and middle pieces. Then secure the center of the springs with the M3 screws. The second way of providing a constant pressure on the finger involves placing 4 M2 screws on the side of the oximeter. The screws act as pulleys for a rubber band or extension spring. In the latter case, the spring tension must be carefully adjusted; the resulting pressure must be light, otherwise the heartbeat will not be detected.

1. Connecting the electronics

Both the sensor and the screen communicate through the I2C standard. Therefore, the SDA and SCL PINs of these electronic components and the corresponding PINs of the ESP32, which are, respectively, 21 and 22, have to be connected for the protocol to work correctly. For both the screen and the sensor, VCC PINs should be connected to the ESP32’s 3.3V PIN, while GND PINs to ESP32’s GND. Then, connect INT PIN on the sensor and ESP32 PIN 34 (which it can be changed at software level if needed).

The easiest way to make the connections is using Dupont cables. However, some soldering may be needed, since some cables (the two SDA, SCL and 3.3V) end on the same ESP32 PINs. In alternative, the cables can be soldered directly on the sensor PINs. ESP32 PINs can be (carefully) bent when more housing space is needed.

After completion and testing of the circuit, cables and junctions can be fixed and reinforced with hot glue, to prevent unwanted disconnections.

1. Programming

We used Arduino IDE to upload the sketch on the ESP32 board. The instructions for program installation and board configuration are available here: <https://randomnerdtutorials.com/installing-the-esp32-board-in-arduino-ide-windows-instructions/>. When choosing the board from the "Tools -> board" menu, "ESP32 Dev Module" is the correct option. Also, a number of libraries are required to have the sketch work correctly. Specifically, it is necessary to download and install:

- Autoconnect (allows connection to the WiFi network). Instructions: <https://hieromon.github.io/AutoConnect/index.html>.

Download Autoconnect: <https://github.com/Hieromon/AutoConnect>,

Pagebuilder: <https://github.com/Hieromon/PageBuilder>,

ArduinoJson <https://github.com/bblanchon/ArduinoJson>.

To install a library, as described in the first link, download the.zip file containing the library. Then, open Arduino IDE, go to "Sketch -> #include library -> add library from .zip file" and the software will install it.

- ESP32 MailClient. Instructions: <https://randomnerdtutorials.com/esp32-send-email-smtp-server-arduino-ide/>, download: <https://github.com/mobizt/ESP32-Mail-Client>
- Adafruit SSD1306 (OLED screen). Download: <https://github.com/adafruit/Adafruit_SSD1306>
- MAX30102_by_RF (signal processing from the sensor). Instructions: <https://www.instructables.com/id/Pulse-Oximeter-With-Much-Improved-Precision/>, download: <https://github.com/aromring/MAX30102_by_RF>.

1. Calibration

The oximeter needs to be calibrated before use. The equation which includes the calibration formula (*pn_spo2 = (-45.060*xy_ratio + 30.354)*xy_ratio + 94.845;) is located in the "algorithm_by_RF.cpp" file within the "MAX30102_by_RF-master" library folder. The formula needs to be updated with values obtained from the calibration procedure to provide meaningful results. Also, some Chinese manufacturers have produced cloned sensors with inverted IR and red LED channels. To account for this problem, the line “xy_ratio= (f_y_ac*f_ir_mean)/(f_x_ac*f_red_mean);” has to be replaced with “xy_ratio= (f_x_ac*f_red_mean)/(f_y_ac*f_ir_mean);”. An updated file with the new equation is already available in the Google Drive folder <https://drive.google.com/open?id=1zvxwnRE-UM87otZBnbQbLkbCB3mnYoJY>. It should replace the original file (located in the Arduino\libraries\MAX30102_by_RF-master folder).

To find the correct values for the calibration equation, thanks to the help of the Maker Giacomo Mandelli, we have developed a streamlined procedure, which is described in details in the "CalibrazioneSaturimetro.pdf" file in the "Calibration" folder.

Essentially, it comes to:

- replacing the files of the MAX30102_by_RF library with those present in the “Calibration” folder;
- Upload the calibration sketch to the oximeter;
- Follow the in-screen instructions. For reaching a low saturation level, just holding the breath may be sufficient. Then, type the saturation values reported by a certified oximeter on the PC keyboard when instructed. The software will take care of the pairing with the home-made oximeter’s values;
- Save the data in a text file;
- Load it in the Fortran program available in the “Calibration program” folder;
- Replace the equation values in the original “algorithm_by_RF.cpp” file with the results and restore the original “algorithm_by_RF.h”;
- Load the “Software.ino” sketch to the ESP32.
